# Advancements in Single-Molecule Fluorescence Detection Techniques and Their Expansive Applications in Drug Discovery and Neuroscience

**DOI:** 10.3390/bios15050283

**Published:** 2025-04-30

**Authors:** Jing Yan, Lin Cheng, Yitong Li, Ru Wang, Jie Wang

**Affiliations:** 1 Department of Veterinary Medicine, University of Cambridge, Cambridge CB3 0ES, UK; 2 Holosensor Medical Technology Ltd., Room 12, No. 1798, Zhonghuayuan West Road, Yushan Town, Suzhou 215000, China; llyn.cheng@holosmed.com (L.C.); ruby.li@holosmed.com (Y.L.); liana.wang@holosmed.com (R.W.); 3 Institute for Advanced Materials, School of Material Science and Engineering, Jiangsu University, Zhenjiang 212013, China

**Keywords:** single-molecule fluorescent probe, biosensor, drug discovery, neuroscience

## Abstract

Single-molecule fluorescence technology stands at the forefront of scientific research as a sophisticated tool, pushing the boundaries of our understanding. This review comprehensively summarizes the technological advancements in single-molecule fluorescence detection, highlighting the latest achievements in the development of single-molecule fluorescent probes, imaging systems, and biosensors. It delves into the applications of these cutting-edge tools in drug discovery and neuroscience research, encompassing the design and monitoring of complex drug delivery systems, the elucidation of pharmacological mechanisms and pharmacokinetics, the intricacies of neuronal signaling and synaptic function, and the molecular underpinnings of neurodegenerative diseases. The exceptional sensitivity demonstrated in these applications underscores the vast potential of single-molecule fluorescence technology in modern biomedical research, heralding its expansion into other scientific domains.

## 1. Introduction

Single-molecule technologies, leveraging their unique capabilities to image, label, and manipulate individual biomolecules, have profoundly transformed biological research by offering unprecedented access to fundamental biological processes that were previously inaccessible or unmeasurable [1]. Unlike classical measurement techniques that detect an average signal over an entire population of molecules, single-molecule detection techniques enable the detection of conformational heterogeneity, the identification of transient or rare conformations, the tracking of conformational changes over time, and the revelation of parallel reaction pathways [2], which have become the cornerstone of leading technologies across various fields, including biophysics, biochemistry, biotechnology, neurobiology, immunology, DNA sequencing, and medical diagnostics [3]. The single-molecule fluorescence detection originated from measurements of singly doped molecules in triphenylene crystals at low temperatures in 1989 and 1990 [4]. The first successful single-molecule fluorescence assay at room temperature was achieved by Betzig and colleagues in 1993 [5]. Two years later, Funatsu et al. demonstrated the imaging of individual fluorescently labeled myosin molecules in aqueous solution [6]. Since then, single-molecule fluorescence technology has undergone rapid evolution and now stands as a vital tool for deciphering complex biological systems [7,8].

The initial crucial step in single-molecule fluorescence detection involves the fluorescent labeling of the target biomolecule. The selection of the fluorescent probe significantly impacts the performance of single-molecule fluorescence detections by influencing the achievable fluorescent intensity and, ultimately, the spatiotemporal resolution [9]. The effectiveness of fluorescent labels hinges on numerous factors, including photostability, label size, environmental sensitivity, labeling efficiency, quantum yield, wavelength, and linker size, among others [10]. Subsequently, the fluorescent signals emitted by the labels on target molecules must be captured, visualized, and quantified using sensors and imaging systems. Advancements in fluorescent dyes/probes, in conjunction with sophisticated imaging and sensor systems, have fueled the rapid progression of single-molecule detection techniques [11]. The latest single-molecule fluorescence sensors and microscopes facilitate multicolor imaging, enabling highly localized tracking of individual molecules at the nanoscale and even extending to three-dimensional space [12,13]. Therefore, in this review, we delve into the remarkable advancements of single-molecule fluorescence detection technologies, organized into three sections: single-molecule fluorescence probes, single-molecule fluorescence imaging systems, and single-molecule fluorescence biosensors. We explore the applications of these technologies in drug discovery and neuroscience and critically analyze the existing challenges and emerging trends in single-molecule detection technologies.

## 2. Single-Molecule Fluorescent Probe

Fluorescent bioprobes serve as potent instruments for analytical sensing and optical imaging, enabling direct visualization of bioanalytes at the molecular level and offering valuable insights into intricate biological structures and processes [14,15]. The selection of a fluorescent probe significantly influences the attainable spatiotemporal resolution and range, ultimately determining the information accessible and thereby profoundly impacting the efficacy of single-molecule experiments [7,9].

**Figure 1:**
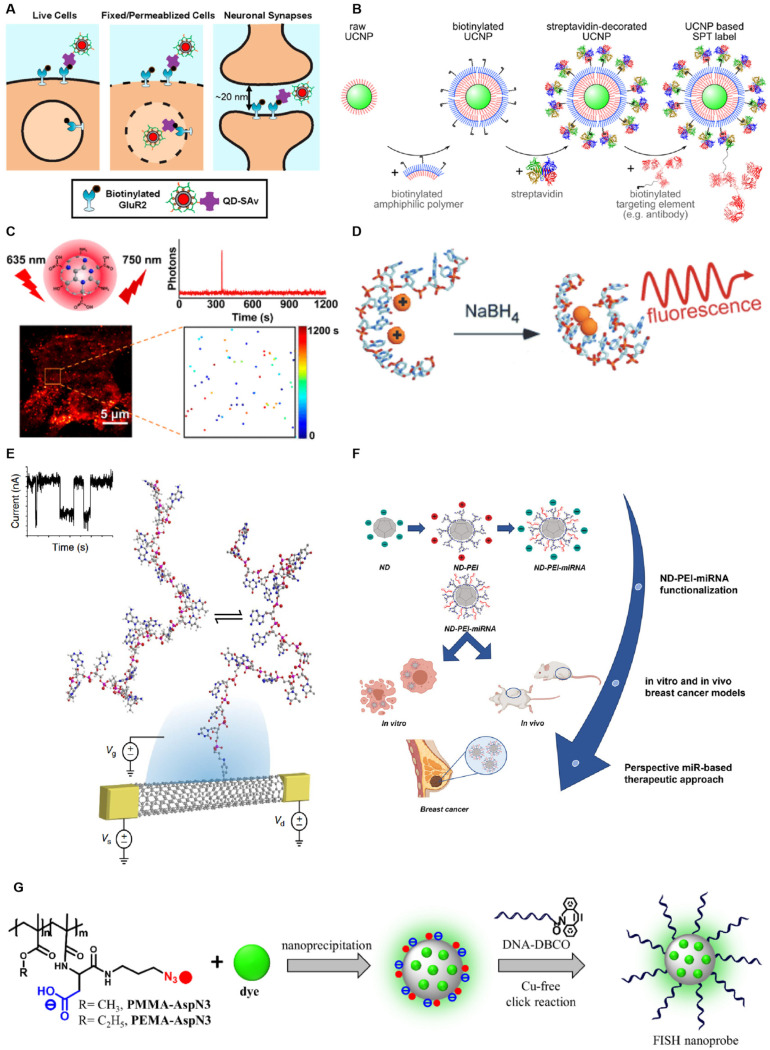
Nanoparticles utilized as single-molecule fluorescent probes. (**A**) Schematic representation of various molecular labeling approaches evaluated using quantum dots of diverse sizes as fluorescent probes. Reprinted with permission from ref. [29]. Copyright © 2020 American Chemical Society. (**B**) A biotin–streptavidin–biotin approach is depicted for the assembly of upconverting fluorescent nanoparticle (UCNP)-based single-particle tracking (SPT) labels. Reprinted with permission from ref. [24]. Copyright © 2024 American Chemical Society. (**C**) Near-infrared blinking carbon dots (CDs) specifically engineered for applications in single-molecule localization microscopy (SMLM). Reprinted with permission from ref. [30]. Copyright © 2022 American Chemical Society. (**D**) Schematic of the IR-Emitting C12-Agn formation, which involves the complexation of C12DNA with silver cations, followed by reduction in the mixture with sodium borohydride (NaBH_4_). Reprinted from ref. [31]. Copyright © 2007 by The National Academy of Sciences of the USA. (**E**) Scaled schematic of a SWCNT-based single-molecule field-effect transistor (smFET) device structure. Reprinted from ref. [32]. (**F**) The functional nucleic acid delivery (FND) system designed for the delivery of microRNA-34a (miR-34a). Reprinted from ref. [33]. (**G**) Nanoprobes based on plasmonic nanoparticles (PNPs). Reprinted with permission from ref. [34]. Copyright © 2021 American Chemical Society.

A diverse array of fluorescent markers is now readily available, encompassing organic fluorophores, nanofluorophores, supramolecular fluorophores, and polymeric fluorophores. The most conventional fluorophores are fluorescent proteins, which possess intrinsic specific labeling with controlled stoichiometry and exhibit superior biocompatibility compared to other probe types, allowing their use in diverse environments such as cells, tissues, yeast, and bacteria [9]. Conventional fluorescent proteins, such as cyan fluorescent protein (CFP), yellow fluorescent protein (YFP), and green fluorescent protein (GFP), suffer from low brightness and photostability and may introduce perturbations in specific biological processes [9]. Organic fluorescent probes, including cyanines, oxazines, coumarins, rhodamines, and BODIPYs, exhibit robust photostability, high spatiotemporal resolution, high quantum yield, and minimal quenching [16,17]. However, nearly all organic fluorophores possess low water solubility, lack cell permeability, and may potentially interfere with the biological system under investigation [18]. Supramolecular fluorophores are self-assembled of supramolecules and fluorophores that can form temporary complexes with the target molecules via non-covalent interactions [19,20]. Although these probes possess high selectivity, sensitivity, and tunability [21], the stability, background signals, and non-specific interactions remain challenges in practice. Compared to organic dyes and fluorescent proteins, nanoparticles exhibit superior photophysical properties when employed as fluorescent probes. In single-molecule fluorescence assays, nanoparticles can function not only as signal reporters but also amplify fluorescence signals with the aid of external fields or serve as efficient energy donors or energy acceptors in energy transfer processes of different sensing strategies [22]. Nanoparticles utilized as single-molecule fluorescent probes usually encompass quantum dots (QDs) [23], upconverting fluorescent nanoparticles (UCNPs) [24], carbon dots (CDs) [25], metal nanoclusters (MNCs) [26], single-wall carbon nanotubes (SWCNTs) [27], fluorescent nanodiamonds (FNDs) [28], and polymeric nanoparticles (PNPs) [22] (Figure 1).

QDs are semiconductor nanocrystals with bright fluorescence, narrow and tunable emission bands, and long-term photostability [35,36]. They comprise metals such as Ag, Cd, Hg, Ln, P, Pb, Se, Te, or Zn at a size of 1–10 nm [37]. By confining the motion of electrons in all three spatial directions, QDs exhibit unique optical and electronic properties [38], helping apply them in single-molecule detection. A quantitative single-molecule localization microscopy technique (qSMLM), which relies on spontaneous blinking, was developed by He et al., enabling the accurate counting of single molecules using QDs as small as 10 nm on a conventional microscope, without the need for complex laser control [39]. Peng et al. constructed templated silver nanocluster-generating molecular beacons (AgNC-MB), a single-molecule counting platform that utilizes QDs in conjunction with AgNC. This platform is activated by probe-target hybridization, followed by a toehold-mediated strand displacement reaction, which releases AgNC-stabilizing sequences and results in fluorescence formation on the DNA template [40]. Compared to conventional organic fluorophores, QDs possess advantages in spectral tunability, quantum yield, and high photostability [41]. However, the application of QDs faces three significant limitations: 1. They lack the photoswitching property crucial for sequential detection and localization in single-molecule imaging. 2. The non-specific and multivalent nature of QDs may induce off-targeting effects, such as oligomerization, activation, internalization, or redistribution of molecules, thereby complicating their use [42]. 3. QDs are larger than commonly used green fluorescent protein markers and organic dyes, and their large size hinders their effective passage through intact cellular membranes.

SWCNTs, composed of atomically thin layers of carbon atoms rolled up into elongated cylinders, belong to a class of optically active nanomaterials. SWCNTs emit near-infrared (NIR) fluorescence, exhibit a large Stokes shift, maintain a constant single-molecule fluorescence intensity over time, and do not scintillate or photobleach under ambient conditions [43,44]. Label-free single-molecule detection of DNA hybridization kinetics has been achieved using SWCNTs [27,32]. However, there are also pressing issues regarding SWCNT production and use. During production, SWCNTs exhibit complex polydispersity in terms of individual chemical composition, lengths, defect densities, and impurity contents. Additionally, dispersion processes, such as sonication for individualized single-walled carbon nanotubes, often lead to the fragmentation of nanotubes, exposing their inner lumen to the solvent. The chemical purification method used for commercial SWCNT materials removes metal-catalyzed particles and carbonaceous impurities but also inadvertently removes 100% of the end caps of the nanotubes [45]. Furthermore, multiple chiralities are present in typical SWCNT samples, complicating the identification of defect-related emission features [46].

The fluorescence of FNDs originates from complexes formed by vacancies and impurities in diamond crystals. FNDs are highly compatible with multimodal imaging techniques, such as stimulated emission depletion microscopy (STED), two-photon microscopy, photoacoustic microscopy, and live cell fluorescence microscopy. Moreover, FNDs with immunolabeling are biocompatible, non-toxic, and can be efficiently internalized within cells through endocytosis [47]. These properties render FNDs particularly suitable for single-molecule detection. However, challenges in the use of FNDs include difficulty in process control, high sensitivity to environmental factors, challenges in modifying their surface for coupling with bioactive agents, and nonspecific accumulation [48,49].

PNPs exhibit efficient energy transfer and anomalous single-particle scintillation, with only one dye molecule per nanoparticle [50]. Egloff et al. encapsulated cyanine and rhodamine dyes with bulky counterions to create green, red, and far-infrared luminescent nanoparticles that are 2–100 times brighter than corresponding quantum dots. Combining these nanoparticles with FISH produced a rapid and sensitive method for single-cell transcriptomics analysis with high sensitivity [34]. However, as polymeric materials, the stochastic synthesis of polymer backbone lengths and the functionalization of hydrophobic and hydrophilic pendant groups result in inevitable structural diversity, making PNPs heterogeneous in nature. This heterogeneity poses a significant challenge for characterization at the single-particle level [51].

Given the complexity of single-molecule detection and data analysis, further efforts are needed to enhance the utility of fluorescent probes for better photon yield and smaller size in the field of single-molecule fluorescence detection. Advancements in probe design, synthesis, and characterization techniques are crucial to overcoming current limitations and expanding the applications of these nanomaterials.

## 3. Single-Molecule Fluorescence Microscopy System

Single-molecule fluorescence imaging techniques can be broadly categorized into two primary areas: one involves studying single-molecule activities when subjected to external forces, typically applied using atomic force microscopy (AFM), optical tweezers (OT), or magnetic tweezers (MT); the other focuses on observing single-molecule activities within biological systems through the use of fluorescence microimaging. Single-molecule fluorescence microscopy (SMFM), which builds upon conventional fluorescence microscopy, enabling the detection and analysis of individual fluorescent molecules employed as reporter labels in biological samples [14], has emerged as a forefront tool for achieving single-molecule fluorescence detection [52]. In 2014, the Nobel Prize in Chemistry was awarded to Eric Betzig, William E. Moerner, and Stefan W. Hell for their invaluable contribution to super-resolution microscopy [53]. This section delves into the specifics of SMFM systems.

### 3.1. Total Internal Reflection Fluorescence Microscopy

**Figure 2:**
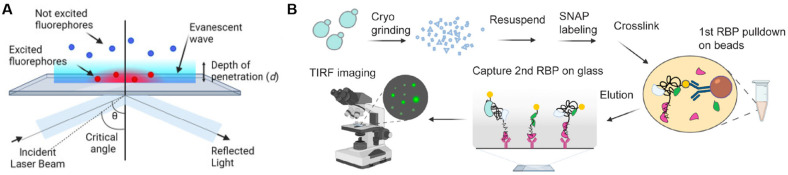
Total internal reflection fluorescence. (**A**) Principle of TIRF. Reprinted from ref. [54]. (**B**) Schematic diagram of mRNP-SiMPull procedure for isolating mRNPs from yeast cells and single-molecule imaging of RBP components. Reprinted from ref. [60].

Total internal reflection fluorescence (TIRF) microscopy stands as one of the most frequently utilized tools in SMFM. It harnesses the principle of total internal reflection to observe molecules in close proximity to an interface through excitation light generated at the junction of a medium with a high refractive index (typically glass) and one with a low refractive index (e.g., sample solution) (Figure 2A) [54]. Besides observing fluorescent molecules within approximately 100 nm of the coverslip, the application of TIRF technology allows for the isolation of individual molecules spaced at intervals exceeding the diffraction limit (about 200 nm) [55]. The evanescent wave generated during total internal reflection decays exponentially with distance from the interface, enabling the excitation of fluorophores close to the surface rather than those that are further away, minimizing background noise [56]. This spatial restriction enhances sensitivity and contrast in imaging. These features make TIRF well suited for single-molecule fluorescence studies and cell biology, such as tracing endocytosis, exocytosis, cell signaling, as well as protein interactions at the cell membrane [57,58]. For instance, Walker et al. reported a TIRF microscopy-based single-molecule photobleaching step analysis technique for studying the oligomeric organization of diverse membrane proteins [59]. The single-molecule pull-down technique, which combines TIRF microscopy with traditional pull-down analysis, facilitates the detection of individual macromolecular complexes directly from cell or tissue extracts. This enables the direct capture and analysis of trace protein quantities in clinical contexts, such as membrane and intracellular antigens in human blood samples [1]. Asada et al. developed a TIRF-based imaging assay (mRNP single-molecule pull-down) to investigate stoichiometry and co-occupancy of mRNPs, as shown in Figure 2B, which achieved quantitative measures of mRNP composition at the single-molecule level [60]. While several limitations remain when applying TIRF into single-molecule studies, such as depth of field, spatial resolution, and photobleaching, studies of chip-based TIRF and waveguide-based TIRF have provided solutions to different extents [61,62].

### 3.2. Stimulated Emission Depletion Microscopy

**Figure 3:**
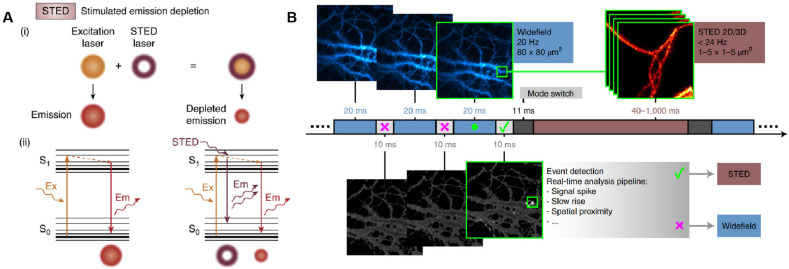
Stimulated emission depletion. (**A**) Schematic diagram of STED nanoscopy. Reprinted from ref. [65]. (**B**) Schematic diagram of event-triggered STED. Reprinted from ref. [69].

Stimulated emission depletion (STED), a technique grounded in confocal laser scanning, attains super-resolution through the incorporation of a high-power STED laser. It operates by depleting fluorophores in an annular ring surrounding the focal point, thereby allowing fluorophores at the focal point to freely emit light upon excitation [63]. That is, the first “excitation beam” focuses on the sample to excite the fluorophores, and the second “depletion beam” then stimulates the emission of fluorophores outside the target region, effectively narrowing the point spread function (PSF) of the fluorescent signal and achieving finer spatial resolution (Figure 3A) [64,65]. STED microscopy offers outstanding performance in bioimaging at the single-molecule level and live cell imaging. Correlation and multimodal approaches that integrate STED microscopy with other detection techniques have further enabled single-molecule detection with enhanced resolution. For instance, combining STED microscopy with AFM allows for nanoscale manipulation via the AFM tip, while the super-resolution localization and visualization capabilities of STED imaging facilitate swift and precise identification of cellular structures at sub-diffraction sizes and manipulation of the AFM tip onto the target [66]. The fusion of STED microscopy with transmission electron microscopy (TEM) offers the highest spatial resolution of plasma membrane proteins and their underlying membranes, which is invaluable for studying cellular events like endocytosis, membrane trafficking, or membrane tension adaptation [67]. While STED can achieve rapid image acquisition without intricate data processing, the spatial resolution it provides is inferior to that of the SMLM technique. Moreover, STED microscopy is also susceptible to photobleaching and photodamage. Combining expansion microscopy with STED has provided an effective spatial resolution below 10 nm in 2D and in the range of 50–70 nm in 3D, although the signal-to-noise ratio was decreased due to the residual STED intensity in the center of the donut, and the inherent photobleaching may have slightly increased [68]. To address these issues, Alvelid et al. presented an event-triggered STED (Figure 3B) that is capable of initiating 2D and 3D STED imaging within 40 ms from detecting cellular events, thus minimizing the overall cell stress and photodamage and maximizing the temporal resolution [69]. Using this tool, they recorded synaptic vesicle dynamics, endocytosis and exocytosis, and the interaction between endosomal vesicles in real time.

### 3.3. Single-Molecule Localization Microscopy

**Figure 4:**
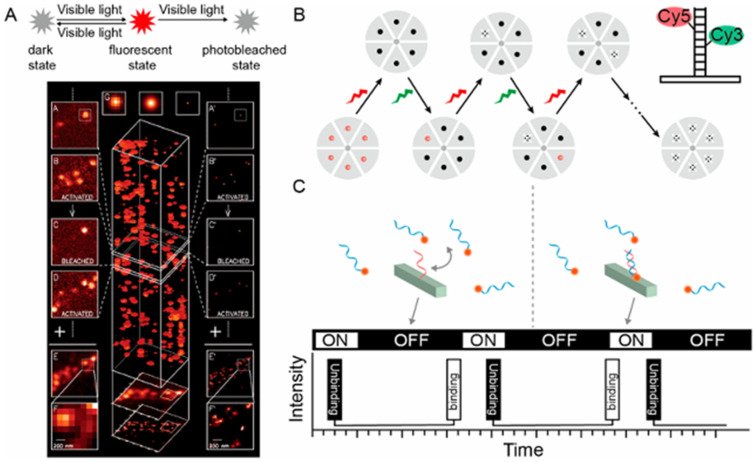
Principles of three single-molecule localization microscopy. (**A**) Principle of PALM. The PA-FP (photoactivatable fluorescent protein) molecules attach to proteins of interest. (**B**) Principle of STORM. Improved clarity by separating the roles of the green and red lasers and refining the description of the imaging process. (**C**) Principles of DNA-PAINT. Reprinted with permission from ref. [76].

Single-molecule localization microscopy (SMLM) describes another family of SMFM techniques that take advantage of precise localization of single fluorescent molecules, offering outstanding spatial resolution and are capable of imaging bio-structures at the molecular scale [70]. Based on the distinct separation mechanisms employed for individual fluorescent molecules, SMLM can be further categorized into three series: (I) photoactivation/switching-based microscopy, which encompasses photo-activated localization microscopy (PALM), stochastic optical reconstruction microscopy (STORM), direct STORM (dSTORM), and ground-state depletion imaging (GSDIM); (II) dynamic labeling microscopy, represented by point accumulation for imaging in nanoscale topography (PAINT) that relies on reversible binding; (III) fluorescence lifetime separation-based microscopy [71]. Figure 4 shows the principles of PALM, STORM, and DNA-PAINT. PALM employs light-activated fluorescent probes to visualize fluorescently labeled molecules with nanoscale precision through iterative imaging and positional accumulation [72]. For PALM in Figure 4A, a 405 nm wavelength laser is employed to activate a fluorescent state, followed by a 561 nm wavelength laser that stimulates fluorescence emission, ultimately leading to bleaching. This sequence is iterated multiple times until all fluorescent molecules are bleached. Subsequently, the accumulated fluorescent signals are merged to pinpoint and reconstruct the image [73]. STORM utilizes a light-induced fluorescent probe to accumulate positions of single molecules by controlling the on-off switching of the probe, thereby achieving ultra-high resolution [74]. As shown in Figure 4B, in each imaging cycle, a green laser initiates the excitation of a subset of fluorescent molecules, whereas a red laser subsequently stimulates these molecules to produce fluorescence until they transition to a dark state. By repeatedly cycling through this process and integrating the precise positions of the collected fluorescent molecules, super-resolution imaging is attained. PAINT integrates the principles of bimolecular collision-induced intermittency, photobleaching, and PSF measurements in a label-free approach for the target object. Post-collisional binding generates a fluorescent signal peak, enabling the use of a virtually endless number of probe molecules to construct nanometer-resolution images [75]. For DNA-PAINT, transient bonding occurs repeatedly between the imager probe, labeled with fluorescent dyes, and the docking strand attached to the imaging target, thereby achieving the “blinking” effect (Figure 4C) [76].

Additionally, SMLM encompasses blinkmicroscopy and spectral position determination microscopy (SPDM). The former leverages the fluctuating emission of single molecules to reconstruct super-resolution images, while the latter typically distinguishes dye molecules by randomly toggling their fluorescence states. Time-series image alignment of these scintillation phenomena permits precise localization of scintillating molecules/particles with nanometer accuracy [77]. Notably, another variety of SMLM known as bleaching/blinking-assisted localization microscopy (BaLM) exploits the inherent bleaching and blinking characteristics of commonly utilized fluorescent probes. Fluorophore bleaching or scintillation events are detected by subtracting consecutive images within the series. Subsequent to image subtraction, fluorescence emission signals from individual fluorophores are identified and localized by fitting the fluorescence intensity distribution to a theoretical Gaussian function [78]. However, each SMLM technique relies on having dyes or fluorophores switch between on and off states. To achieve multicolor SMLM, STORM offers controlled photoactivation of the same emitter dye paired with different activator dyes, which precludes chromatic aberration between color channels. Sequential SMLM of spectrally well-separated probes achieves lower crosstalk; exciting with different lasers using a multi-band filter set provides great filter-switching and channel alignment [79]. For 3D imaging, a common solution is to encode z into the shape of a single molecule image, or the PSF of the microscope system. For instance, Velas et al. employed a 3D-SMLM method, combining supercritical angle fluorescence microscopy with defocused imaging, to achieve an isotropic localization precision below 15 nm [80].

TIRF, due to its technical mechanism, has enhanced signal-to-noise ratios ideal for live-cell imaging of interactions at cell membranes. However, it can only probe molecules within approximately 100 nm of the surface. STED combines focused excitation and a depletion beam to achieve super-resolution, allowing for spatial resolution down to 20–50 nm, making it suitable for detailed structural studies of cellular components. Its complexity and high laser power requirements can lead to phototoxicity, limiting its use in live-cell scenarios. In contrast, SMLM relies on stochastic activation and localization of individual fluorophores, achieving exceptional spatial resolution (10–20 nm) by reconstructing molecular positions over time. While SMLM excels in mapping protein distributions and investigating cellular architecture with high precision, it necessitates specific fluorophores and longer acquisition times, which can hinder the analysis of dynamic processes.

In recent years, single-molecule fluorescence imaging techniques have undergone significant updates and refinements. For instance, the advent of MINFLUX, subsequent to STED and ALM/STORM, not only elevates the resolution of fluorescence microscopy to the single-molecule level but also narrows the general resolution disparity of STED, PALM/STORM, and other fluorescent nanoscopes from 20 to 30 nm down to the molecular scale of 1–5 nm [81]. MINFLUX was first commercially introduced in December 2019, albeit its adoption is still limited [65]. The multi-view reflector microscope, proposed by Zhang et al., integrates polarization modulation and pupil-splitting techniques, enabling 6D imaging of fluorescent molecules in single-molecule orientation-localization microscopy (SMOLM) [82]. Recent publications have unveiled a freestanding bilayer microscope (FBM), which merges the merits of freestanding bilayer architecture with single-particle tracking (SPT), facilitating the investigation of membrane protein dynamics with single-molecule resolution and unimpeded diffusion [83]. Zero-mode waveguides have also found widespread application in single-molecule fluorescence detection, capable of achieving single-molecule resolution even in solutions with high concentrations of fluorescent components [84]. Most recently, bond-selective fluorescence-detected infrared-excited (BonFIRE) spectral microscopy was developed, leveraging mid- and near-infrared two-photon excitation to detect vibrational excitation that is upconverted to an electronic state, thereby encoding vibrational information into fluorescence. This system boasts high sensitivity, biocompatibility, and robustness, utilizing tunable narrowband picosecond pulses for bond-selective biodetection of diverse reporter molecules [85].

Various single-molecule fluorescence imaging techniques possess their unique strengths and limitations, and no single microscopy method excels in all aspects, such as achieving high spatial and temporal resolution, minimizing sample irradiance and phototoxicity, and ensuring high image contrast. In some instances, these requirements are inherently conflicting. Consequently, when designing an experiment, it is imperative to select a technique that can effectively address the core biological inquiry. Furthermore, the integration of diverse single-molecule fluorescence detection modules, alongside correlation methods with other microscopy and spectroscopy techniques, presents additional avenues for exploration [86]. Future advancements will necessitate ongoing enhancements in excitation and detection methodologies, fluorescent probe design, synthesis, and delivery techniques, as well as innovative developments in algorithms and imaging techniques.

## 4. Single-Molecule Fluorescent Biosensors

Advancements in single-molecule fluorescence detection technology have significantly contributed to the evolution of ultrasensitive biosensors. These biosensors help realize real-time detection of single molecules with high specificity and accuracy, uncovering unique properties hidden among the molecule crowd, enabling single-molecule tracking, multicolor imaging, and monitoring of biological events at the single-molecule level [13]. This section underscores the endeavors and recent milestones achieved in the realm of single-molecule fluorescent biosensors.

**Figure 5:**
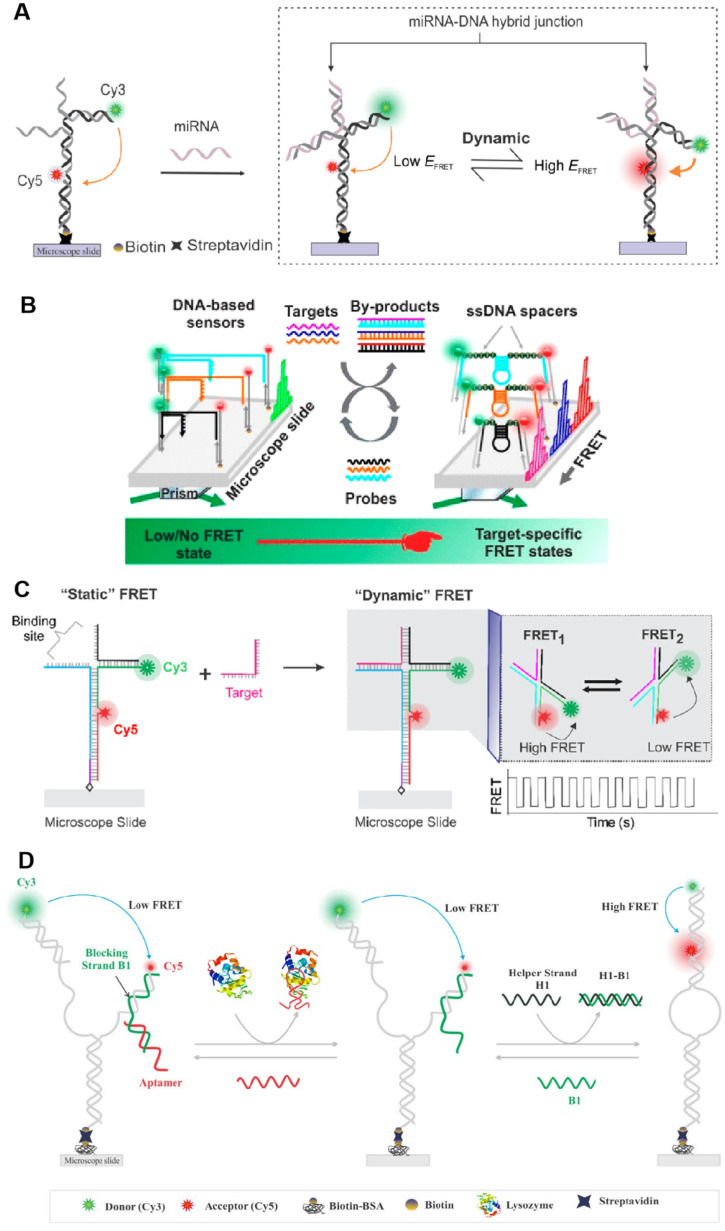
Single-molecule fluorescent biosensors based on the FRET principle. (**A**) A DNA-based smFRET sensor for high-confidence detection of miRNAs. Reprinted with permission from ref. [90]. Copyright © 2022 American Chemical Society. (**B**) Principles of nucleic acid detection using the interconvertible hairpin-based sensor of smFRET. Reprinted with permission from ref. [91]. Copyright © 2019 American Chemical Society. (**C**) Working principle of a smFRET-based dynamic DNA sensor. Reprinted with permission from ref. [92]. Copyright © 2021 American Chemical Society. (**D**) SmFRET-based aptamer sensors detect lysozyme selectively and sensitively. Reprinted from ref. [95].

Based on their functionality, there exist numerous types of single-molecule fluorescent biosensors. Notably, these biosensors have been devised utilizing the principle of single-molecule fluorescent counting and single-molecule Förster (or fluorescence) resonance energy transfer (FRET). FRET involves the non-radiative transfer of energy from an excited donor fluorophore to an adjacent acceptor fluorophore, prompting the acceptor to emit fluorescence at its characteristic wavelength [87]. The pioneering, genetically encodable, single-molecule FRET biosensor, Cameleon, was introduced in 1997, enabling the monitoring of local Ca^2+^ signaling and protein heterodimerization within a single living cell [88]. Since its inception, a spectrum of single-molecule fluorescent biosensors based on the FRET principle has been developed, yielding groundbreaking results that facilitate real-time monitoring of DNA synthesis [89], quantification of miRNA and nucleic acid biomarkers [90,91], identification of diverse nucleic acid sequences [92], rapid kinetic profiling of individual nucleic acid molecules [93], visualization and quantification of structural dynamics in single-protein biomolecules [94], and selective and sensitive detection of lysozyme [95], among others (Figure 5).

The production of single-molecule fluorescent biosensors necessitates three pivotal components: high detection sensitivity, selectivity, and preferably precise localization. Recent advancements underscore the utilization of innovative nanomaterials and microfluidics in the development of single-molecule biosensors, significantly enhancing their detection sensitivity [13]. Xu et al. seamlessly integrated lanthanide complexes, FRET, QDs, and rolling circle amplification (RCA) into a microRNA biosensor, achieving ultra-high sensitivity in detecting individual miRNAs [96]. Pei et al. devised an FNP-based single-molecule fluorescent nanosensor capable of simultaneously detecting three single nucleotide variants (SNVs) [97]. Liu et al. introduced a QD-based smFRET biosensor, which facilitates uniform detection of TET2 with a limit of detection (LOD) of 0.042 ng/μL, accurately quantifying cellular TET2 levels in as few as 1 cell [98]. Huang et al. developed a mitochondria-targeted ratiometric fluorescent nanosensor, employing mitochondria-specific single fluorescent probes for concurrent sensing and imaging of pH and oxygen levels within mitochondria. These probes encapsulate CdS/ZnS QDs in silica housings, serving as internal reference points and carriers for assembling oxygen- and pH-responsive elements with mitochondria-targeted molecules [99]. A novel catalytic QD FRET nanosensor, utilizing a single probe, has been reported, achieving specific sensing and efficient signal amplification. Each signaling probe contains multiple Cy5 molecules, and multiple probes can cluster on a single QD, thereby dramatically boosting FRET efficiency [100]. Krainer et al. presented a surface-free single-molecule microfluidic sensing platform, combining microchip electrophoresis separation with single-molecule fluorescence detection—digital immunosensor assay (DigitISA). This platform has been applied to various targets, including amyloid aggregates, exosomes, and biomolecular condensates, providing information beyond stoichiometric interactions and enabling the characterization of immunochemistry, binding affinity, and protein biomarker abundance [101]. These reports underscore the immense potential of single-molecule fluorescence sensors, benefiting from emerging techniques and their integration in clinical diagnosis and drug discovery. However, for non-fluorescent or weakly fluorescent molecules, fluorescence labeling is required for signal amplification in these biosensors, which could also influence the photophysical properties of fluorescent probes. Therefore, the development of novel probes is essential to lengthen their life span [13]. Meanwhile, efforts in the development of user-friendly and automated instruments, the discovery of more novel fluorescent labels and labeling modalities, and the introduction of advanced concepts will help the improvements of new biosensors [4].

## 5. Applications of Single-Molecule Fluorescence Detection in Drug Discovery and Neuroscience

Since their introduction, single-molecule fluorescence measurements have been employed to tackle numerous challenges across diverse fields. This chapter specifically delves into the applications of single-molecule fluorescence detection techniques within the realms of drug discovery and neuroscience.

### 5.1. Drug Discovery

Continuous theoretical advancements and technological refinements in single-molecule fluorescence assays have significantly contributed to the design and monitoring of intricate drug delivery systems. In contrast to traditional surface-based techniques, these techniques boast low sample consumption and enable highly sensitive determination of affinity, particularly for weakly bound biomolecules [102]. Through real-time observation of single-molecule behaviors, molecular interactions, and dynamics, single-molecule fluorescent techniques offer insights into detailed mechanisms of diseases, novel drug targets, and potential drug screening.

SMFM stands out as a pivotal technique for elucidating the behavior of nanomaterials tailored for drug delivery studies [103]. Belfiore et al. utilized single-molecule fluorescence imaging to quantitatively assess the content of targeting molecules on functionalized liposomes, notably plasminogen activator inhibitor-2 (PAI-2) and trastuzumab (TZ, Herceptin^®^), targeting cancer cell surface biomarkers, benefiting the development of targeted liposomal drug delivery systems [104]. The developed single-molecule approach is also expected to be useful for the quantification of antibody fragments, small peptides, and aptamers and holds potential for large-scale manufacturing processes and batch-to-batch quality control in commercial settings. Paul Joyce et al. pioneered the use of TIRF microscopy to monitor temporal alterations in the adsorption and diffusion of biologically active drug compounds from the bulk phase into the pores of mesoporous silica membranes and through supported cellular membrane mimics, thereby directly evaluating in vitro drug penetration across model lipid membranes [105]. Using this platform, the C8-induced permeability of a model drug in oral delivery conditions can be monitored and validated, and potential applications for optimizing small molecule bioactive permeation across widespread lipid membrane barriers are expected [105]. Prabhakar et al. described how a novel composite comprising a photoluminescent nanodiamond core and a porous silicon dioxide (SiO_2_) shell can be harnessed for single-molecule fluorescence-based STEDs, probing bioimaging and drug delivery applications [106]. Carboxypolystyrene (PS) nanofluorescent particles are extensively used in drug delivery due to their excellent biocompatibility, straightforward synthesis, and robust stability. Wang et al. conducted fluorescence-based single-molecule tracking using confocal microscopy to reveal the intracellular transport of drug-laden PS nanoparticles [107]. In a combination of dSTORM and electron microscopy (CLEM), Andrian et al. traced nanoparticles along the endolysosomal pathway at various time points and demonstrated the impact of chloroquine on the intracellular distribution of nanoparticles (i.e., endolysosomal escape) [108]. Utilizing dSTROM, Kota et al. observed that zeolitic imidazolate framework-8 (ZIF-8) nanoparticle drug carriers induced changes in cell morphology and actin cytoskeleton organization on both apical and basolateral surfaces of cells, thereby disrupting the contractile function of vascular endothelial cells in response to angiotensin II, even at concentrations deemed “safe” by biochemical cytometry [109].

SMFM can further be leveraged in pharmacokinetic monitoring and the exploration of pharmacological actions and mechanisms. For instance, Yanagawa and Sako previously provided a comprehensive workflow for measuring the kinetics of drug activation of G protein-coupled receptors (GPCRs) and metabotropic glutamate receptor 3 (mGluR3) using TIRF microscopy [110]. The case study presented by Wang underscores the prowess of smFRET as a tool for guiding the exploration of drug mechanisms of action, revealing that neomycin exhibits pleiotropic effects on the translational machinery as a direct consequence of its binding to H69 of the large subunit, whose presence inhibits the relative movements of the small and large subunits necessary for function [111]. The modulation of nuclear size by nocodazole and paclitaxel was previously observed using STED [112]. STED microscopy also assisted Beri et al. in capturing high-resolution images of drug-treated parasites, reflecting the correlation between morphological changes and specific metabolic blockages [113]. Conze et al. employed DNA-PAINT to determine the effect of the microtubule-targeting drug MTA epothilone D (EpoD) on microtubule arrays at concentrations promoting increased microtubule polymerization [114]. Leveraging the SMLM platform, Weinelt et al. also demonstrated and confirmed that zafirlukast inhibits ligand-independent TNFR1 PLAD-PLAD-mediated dimerization and TNFα-induced higher-order TNFR1 aggregation [115]. Hartley et al., in their study, exhibited how dSTORM imaging can be utilized to assess drug release from the carrier and observed alterations in membrane organization following exposure to therapeutic drugs using dSTORM [116]. PALM was previously employed to quantitatively characterize the differential effects of pharmacologically relevant concentrations of ethanol and naltrexone, an opioid receptor antagonist and drug used to treat alcohol use disorders, on the lateral nano-organization of mu and kappa opioid receptors (MOR and KOR, respectively). The results demonstrated that ethanol reduced the size and occupancy of opioid receptor nanodomains and increased the proportion of opioid receptors located outside the nanodomains, while naltrexone significantly increased the surface density, nanodomain size, and occupancy of KOR, exerting minimal influence on these MOR parameters [117].

In addition to SMFM, single-molecule-based fluorescence correlation spectroscopy (FCS) and FRET have proven especially useful in the field of pharmacological research and drug discovery. Yadav et al. demonstrated a direct effect of the broad-spectrum antibiotic chloramphenicol on the passive elasticity of the muscle protein Titin, utilizing both single-molecule force spectroscopy and fluorescence spectroscopy [118]. Over the past decade, numerous efforts have successfully integrated single-molecule FRET (smFRET) into drug discovery experiments. Georg Krainer and Antoine Treff, among others, used smFRET to investigate the misfolding and pharmacological rescue mechanism of the cystic fibrosis transmembrane conductance regulator (CFTR). Their findings revealed that the V232D hairpin exhibits an open conformation in the lipid bilayer, which is reversed by the pharmacological corrector Lumacaftor [119]. The newly reported smFRET platform is also capable of detecting protein misfolding and reading out the FRET efficiency with precision [120]. A study by Jiawei Zhao and Matthias Elgeti, among others, employing fluorescent labeling of intracellular residues and the smFRET platform to investigate the µ-opioid receptor, found that conformational changes in this receptor correlate with ligand efficacy, both in the presence and absence of different ligands bound by G proteins [121].

However, challenges such as photobleaching and complex data analysis hinder broader application of single-molecule fluorescence techniques-based drug discovery in clinical and industrial settings. Future developments should focus on improving fluorescent probes, advancing imaging technologies, leveraging machine learning for data interpretation, and integrating with other modalities for better elucidating drug-target interactions and screening novel compounds in complex biological systems. The aforementioned assays provide practical research tools for the drug discovery process from diverse perspectives. Indeed, the opportunities presented by single-molecule fluorescence technologies to unravel typical scientific challenges in drug discovery are numerous and growing exponentially. It is anticipated that the development of entirely novel tools and protocols will significantly propel drug discovery efforts in the near future.

### 5.2. Neuroscience

Neuroscience examines the electrophysiology of neurons and synapses, micro- and macro-neuroanatomy, and the functional organization of brain regions [122]. The physical dimensions of cellular components of the brain, such as intra-neuronal, presynaptic, and postsynaptic structures, may be less than a few hundred nanometers, thus necessitating microscopes with nanoscale resolution [123,124]. In neuroscience, SMFM has aided in understanding synaptic and general neuronal function, offering fresh insights into previously unrecognized phenomena and resulting in groundbreaking discoveries. Single-molecule fluorescence approaches have expanded our knowledge of RNA movement and localization within living neurons [125]. For instance, single-molecule fluorescence imaging of endogenous β-actin mRNA in neurons has confirmed the sequential assembly and disassembly of large mRNP complexes containing multiple copies of β-actin mRNA. Furthermore, increased β-actin expression may be linked to the transduction of synaptic activity into structural plasticity [126]. Thanks to advancements in single-molecule fluorescence techniques, the translation kinetics of individual mRNAs in living neurons have been observed. In primary neurons, mRNAs are translated in proximal dendrites but repressed in distal dendrites, exhibiting a phenomenon known as “burst” translation [127]. Imaging β-Actin mRNA with single-molecule sensitivity revealed that most β-Actin mRNA molecules are present independently in growing axons. While improvements in RNA detection in neurons may inevitably introduce discrepancies with previously published reports, these findings will ultimately enrich our understanding of local protein synthesis in neurons.

**Figure 6:**
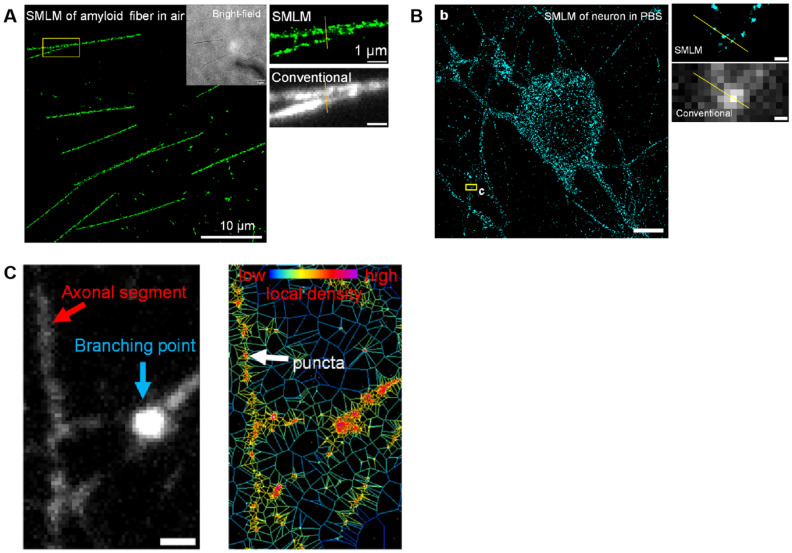
SMLM bioimages based on burst-blinking nanographenes. (**A**) SMLM of amyloid fiber in air. (**B**) SMLM of neuron in PBS. (b) Reconstructed SMLM image of global nascent proteins in neurons. Imaging was performed in PBS solution. (c) Magnification of SMLM image and conventional wide-field fluorescence image for the yellow box region in (b), respectively. (**C**) Conventional wide-field fluorescence image of networks in neurons (**left**) and corresponding Voronoi diagram image (**right**) of the same position. Reprinted from ref. [133].

Single-molecule fluorescence methods have also deepened our understanding of neural network morphology and structure. According to super-resolution imaging (SMLM) observations, the presynaptic terminal contains distinct actin nanostructures: an actin meshwork defined as the active zone, an actin track between the active zone and the deep reserve pool, and an actin fence surrounding the entire presynaptic compartment [128]. PALM imaging and single-molecule tracking measurements have revealed actin dynamics within the micrometer-sized, single-living dendritic spines. These observations clearly demonstrate that the spinous process contains a dense and highly dynamic perisynaptic actin network, with tightly positioned focal aggregates both inside and outside the synapse, a dense central core with heterofilament orientation, and actin filaments at the neck of the spinous process, frequently featuring barbed ends directed towards the dendritic axis [129]. Direct three-dimensional (3D) SMLM has also revealed the morphology of the immune synapse and cleft size within the synapse, with an isotropic accuracy below 15 nm [80]. The STORM technique has enabled the characterization of the periodic structure of the axonal cytoskeleton and the spatial grouping of synaptic proteins in neurons [130,131]. Potential mechanisms regulating synapses have also been identified in studies using STORM, such as the isolation of bassoon tubes from Cav2.1 and the activity-dependent depolymerization of RIM clusters in hippocampal synapses, accompanied by the recruitment of active zone motifs enriched with bassoon tube structural domains [132]. The special nanographene-based fluorophores for SMLM were leveraged to image amyloid fibrils and lysosome dynamics, achieving single-molecule labeling of nascent proteins in primary sensory neurons (Figure 6). This unique SMLM analysis enabled a detailed map of translationally active foci in neuronal axons at the single-molecule level, helping better understand local translation in response to internal and external stimuli [133].

Neurodegenerative diseases, including Alzheimer’s disease (AD), Parkinson’s disease (PD), and Huntington’s disease (HD), are characterized by the misfolding and aggregation of proteins within the brain. This process results in neuronal death and the subsequent loss of cognitive function. The proteins implicated in each of these disorders—tau and Aβ for AD, α-synuclein for PD, and polyQ-Huntington’s for HD—share striking structural similarities, adopting a β-sheet-rich fibrillar structure known as amyloid [134,135]. Enhanced single-molecule fluorescence detection techniques have been successfully employed to characterize amyloid aggregation in neurodegenerative diseases, investigate the dynamics and mechanisms underlying amyloid formation [136], and delineate the multiple intermediate states present during the lag phase of amyloidogenesis [137]. For instance, aggregation-induced emission is proved for visualization of amyloid fibrils by TRIF microscopy: AIE dyes bind to stacked β-sheet structures, and the signal increases with β-sheet structure, indicating the formation of amyloid fibrils [137]. The oligomer growth kinetics of the SH_3_ structural domain of α-spectrin have been meticulously characterized, affirming the dynamic equilibrium behavior associated with the conversion of oligomers into mature protofibers [138]. SmFRET (single-molecule Förster resonance energy transfer) analysis indicates that the binding of α-synuclein to amphipathic small molecules or membrane chaperones modulates conformational transitions between its native unfolded state and various α-helical structures [139]. Similarly, SmFRET has provided insights into conformational changes in tau protein in Alzheimer’s disease, revealing that different structural domains of tau exhibit distinct conformational properties that are intimately linked to the progression of the disorder [140]. Furthermore, SmFRET-based studies have demonstrated that monomeric Httex1 adopts a tadpole-like structure for polyQ lengths both below and above the pathological thresholds associated with HD. These studies infer that unavoidable higher-order homotypic and/or heterotypic interactions within different neuronal subpopulations, at limited cellular concentrations, may be the primary cause of the sharp polyQ length dependence observed in HD [141].

**Figure 7:**
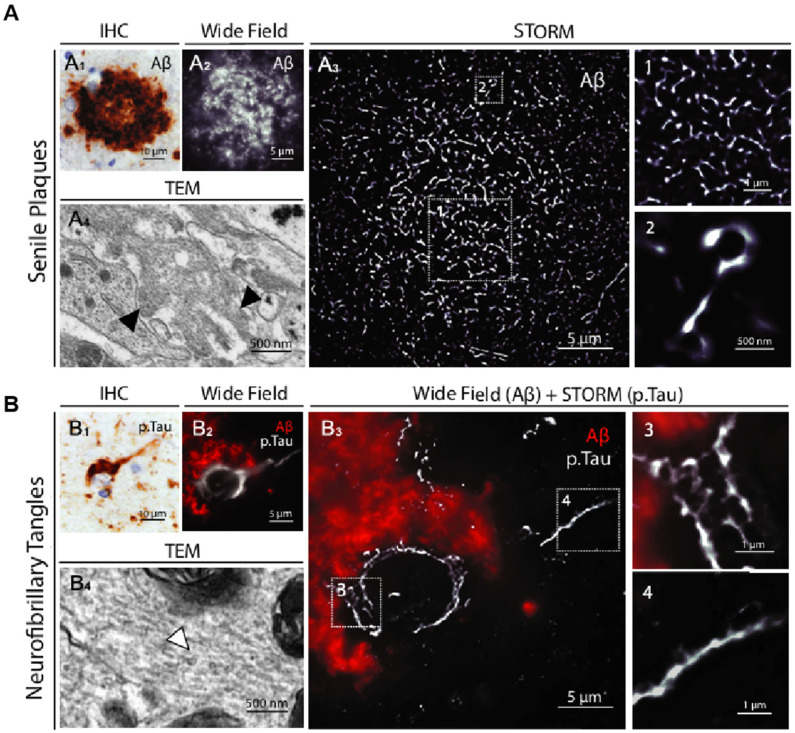
STORM images of senile plaques and neurofibrillary tangles in AD patient brains. (**A**) Shown from left to right are immunohistochemical staining, conventional fluorescence microscopy imaging, and super-resolution STORM images of a senile plaque located in the neocortex of an Alzheimer’s disease (AD) patient. (A_1_) Representative image of a senile plaque in the neocortex of an AD patient (immunohistochemical detection of Ab). (A_2_) Conventional fluorescence microscopy image of a whole senile plaque in a neocortex section of the same patient immunostained for Ab. (A_3_) STORM image of the same area. The insets (1 and 2) show close-up details of the distribution and size of aggregated Ab branches. (A_4_) Comparative TEM image of Ab fibrils (black arrowheads) in a senile plaque. (**B**) Displayed from left to right are representative images of neurofibrillary tangles in the neocortex of AD patients, captured using immunohistochemical staining, conventional fluorescence microscopy, and a combined approach that integrates conventional fluorescence microscopy for β-amyloid (Aβ) detection with STORM imaging for phosphorylated tau (p.Tau) visualization. Reprinted from ref. (B_1_) Representative image of neurofibrillary tangles in the neocortex of an AD patient (immunohistochemical detection of p.Tau). (B_2_) Conventional fluorescence microscopy image of neurofibrillary tangles within the soma of a whole degenerating neuron surrounded by Ab deposition in a neocortex section of the same patient. (B_3_) Same neuron imaged by combining conventional fluorescence microscopy (Ab) and STORM (p.Tau). The insets (3 and 4) show close-up details of the honeycombed structure of p.Tau aggregates in the soma and the filamentous organization in the axon. (B_4_) Comparative TEM image of Tau filaments (white arrowhead) in neurofibrillary tangles [145].

Highly sensitive single-molecule fluorescence detection studies of AD have also illuminated the mechanisms underlying the aggregation and deaggregation of Aβ (1–40) [142]. Specifically, STORM (stochastic optical reconstruction microscopy) in AD has revealed the propagation of AD-associated tau pathology. Exposure to monomeric extracellular tau, even for brief periods, can lead to the infection of healthy cells and initiate the nucleation of aggregate seeds. These seeds bind and rapidly accelerate the aggregation of endogenous tau, and the resulting aggregates are released into the extracellular medium, capable of infecting other cells. This confirms the crucial role of endocytosis in the propagation of AD pathology [143]. A study of PD using SMLM (single-molecule localization microscopy) found that exogenously added monomeric α-synuclein induced the formation of intracellular aggregates and led to apoptosis. This effect was significantly mitigated by preformed protofibrils, thereby confirming that protofibrillar species possess neuroprotective properties [144]. As shown in Figure 7, STORM images provided the highest level of clarity and offered a comprehensive view of the neuropathological hallmarks of AD and PD diseases. The STORM images highlight the unique internal organization and intricate structure of the protein aggregates, revealing details not discernible through conventional methods [145].

It is unequivocally evident that single-molecule fluorescence techniques have significantly advanced neuroscience research endeavors. However, this burgeoning field still harbors numerous unresolved inquiries, such as elucidating the intricate interactions of individual amyloid proteins with cellular mechanisms and identifying which of these proteins are culpable for cell death. The ongoing evolution of single-molecule fluorescence techniques holds the promise of furnishing definitive answers to these pressing questions, unveiling the underlying molecular phenomena that propel these pathologies, and potentially heralding novel therapeutic strategies for the future. However, studies in live neuronal systems at the single-molecule level are limited by challenges such as photobleaching and suitable probes. Additionally, the complexity of neural networks and the vast amount of data generated necessitate advanced analytical methods for effective interpretation. Future developments should aim to enhance the stability and specificity of fluorescent probes, improve imaging technologies for live-cell applications, and integrate machine learning for data analysis to enhance our understanding of synaptic mechanisms and neuronal signaling, ultimately advancing the field of neuroscience.

## 6. Conclusions

In summary, single-molecule fluorescence detection has emerged as a formidable tool in diverse domains, including biology, medicine, and pharmacy, among others. With the relentless progression and refinement of single-molecule fluorescent probes, fluorescent microscopes, and fluorescent biosensors, scientists are now empowered to capture and observe the nanostructures and dynamic behaviors of individual molecules with unprecedented precision.

In realms such as drug discovery and neuroscience, the impact of single-molecule fluorescence technology has been nothing short of phenomenal, demonstrating its extensive applicability in medical research, as well as in the design and monitoring of intricate drug developments. This technology has profoundly accelerated biomedical research and enhanced the specificity and efficacy of drug discovery and therapeutic interventions. In neuroscience, single-molecule fluorescence techniques have been instrumental in unraveling the complexity of neuronal signaling, synaptic function, and the molecular underpinnings of neurodegenerative diseases. These advancements have not only broadened our comprehension of brain function but have also laid the groundwork for innovative diagnostic and therapeutic approaches.

Despite its immense potential, single-molecule fluorescence techniques currently confront challenges, particularly when applied to more intricate and dynamic biological systems. Overcoming limitations associated with signal-to-noise ratio, photobleaching, and real-time imaging within organisms is paramount for further advancements. Moreover, integrating single-molecule fluorescence techniques with complementary technologies, such as artificial intelligence and microfluidics, promises to broaden their application across scientific disciplines. It is anticipated that with the sustained development of single-molecule fluorescence technology, its applications may extend into fields such as clinical diagnosis, personalized medicine, and environmental monitoring, thereby establishing single-molecule fluorescence as the linchpin of modern biomedical research.

## Data Availability

No new data were created or analyzed in this study. Data sharing is not applicable to this article.
